# Macroscopic Singlet-Triplet Qubit in Synthetic Spin-One Chain in Semiconductor Nanowires

**DOI:** 10.1038/s41598-017-05655-9

**Published:** 2017-07-17

**Authors:** Blazej Jaworowski, Nick Rogers, Marek Grabowski, Pawel Hawrylak

**Affiliations:** 1 Department of Physics, University of Ottawa, Ottawa, K1N 6N5 Canada; 2 Department of Physics, University of Colorado, Colorado Springs, CO 80918 USA; 3 Department of Theoretical Physics, Faculty of Fundamental Problems of Technology, Wroclaw University of Science and Technology, Wroclaw, Poland

## Abstract

We show here how to create macroscopic quantum states in a semiconductor device: a chain of InAs quantum dots embedded in an InP nanowire. Filling the nanowire with 4 electrons per dot creates a synthetic spin-one chain, with four-fold degenerate topological ground state protected by a Haldane gap. The four states correspond to two spin-½ quasiparticles localised at the ends of the macroscopic wire. The quasiparticle spins are mapped onto a robust, macroscopic, singlet-triplet qubit. These predictions are supported by a microscopic theory and extensive numerical simulations.

## Introduction

There is currently a great interest in developing solid state quantum information processing devices^1–9^. Some of the most successful, robust and scalable devices rely on qubits built with superconducting macroscopic quantum states^5–7^. Here we propose to generate robust macroscopic quantum states, and a macroscopic qubit, in a semiconductor device realizing a synthetic Haldane spin-one chain^10–12^.

The ground state of spin one antiferromagnetic Heisenberg chain is a topological phase of matter known as the Haldane phase^10–12^. Topological phases, in general, are characterized by the existence of edge states, which are “topologically protected”, i.e., robust to perturbations. In Haldane phase, there are four such states (two at each end of the chain), and despite the fact that the whole chain is made of spins one, these states behave as two effective spins-1/2. They can be understood in the valence bond picture (AKLT state)^11^, in which every spin-1 is a triplet subspace of two virtual spins-1/2. The virtual spins at neighbouring sites are connected with singlet bonds, leaving unpaired spins-1/2 at each end. The resulting four states are ground states, protected from higher energy excitations by the Haldane energy gap. These predictions were confirmed by numerical calculations^13–18^ and experimental studies^19–21^ in quasi-one-dimensional complex compounds. Since then, quantum spin systems have been used as model systems to study macroscopic quantum phenomena^22, 23^.

**Figure 1:**
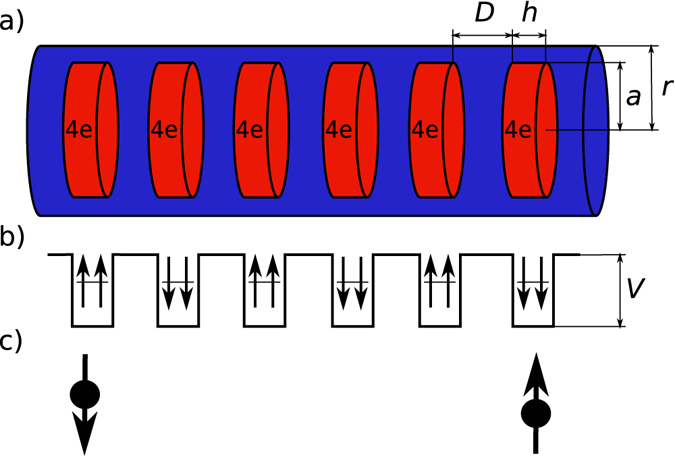
Schematic view of operation of a singlet-triplet qubit in a synthetic spin-one chain realized in an array of nanowire quantum dots. The dots are loaded with 4 electrons each, forming a spin-one ground state due to Hund’s rules. The spin-one dots interact with each other antiferromagnetically via an effective Heisenberg Hamiltonian. The ground state of Haldane spin-one chain is highly correlated, with properties similar to two spin ½ quasielectrons localized on the two ends of the chain.

In this report, we demonstrate that a synthetic spin-one chain can be realized in an array of InAs quantum dots (QD) embedded in a semiconductor, e.g., InP, nanowire^24, 25^. Optical spectroscopy of individual either InAs or InAsP quantum dots in InP nanowire shows existence of electronic s, p, and d shells^24^. It has been shown that using external gate InAs quantum dots can be loaded with a controlled number N_e_ of electrons^26, 27^. With N_e_ = 4, two of the electrons occupy the s-shell and the remaining two electrons occupy the two degenerate orbitals of the p-shell^28, 29^. We show here that as in lens shaped InAs self-assembled quantum dots^28–32^, in InAs quantum dots in InP nanowires exchange interaction of the two electrons on a p-shell leads to a spin polarized, S = 1, triplet ground state. With triplet ground state with total spin S = 1, we propose to use each quantum dot as a building block of a synthetic spin-one Heisenberg chain, in which the hybridization of single-particle levels due to tunnelling leads to effective ferromagnetic interaction. The architecture of the proposed linear spin-one chain in a semiconductor nanowire is shown in Fig. 1a. The device consists of an array of InAs or InAsP quantum dots embedded in InP semiconductor nanowire covered by InP shell. Each quantum dot contains N_e_ = 4 electrons. The electrons are confined by a conduction band offset between strained InAs and InP. We will show that a single quantum dot in a nanowire with N_e_ = 4 electrons has indeed triplet, S = 1, ground state, with two electron spins effectively locked parallel on each dot, as shown schematically in Fig. 1b. Exact diagonalization of a microscopic Hamiltonian shows that the p-shell electrons on neighbouring dots interact with each other leading to effective spin-spin interaction. When the low energy states of a chain of such quantum dots are determined, the ground state is found to be represented by fractional, spin-1/2, quasi-particles localized at the edges of a macroscopic chain as illustrated in Fig. 1c. We next show how the two spin-1/2 quasiparticles can be used to encode and manipulate a macroscopic singlet-triplet qubit^1–4^.

## The microscopic model

We now turn to a theoretical model of our macroscopic singlet-triplet qubit in a synthetic spin-one chain shown in Fig. 1. We start with a single particle spectrum. Extensive atomistic calculations of InAs quantum dots indicate that the effective mass approximation works well for conduction band electrons^32^. We hence describe a single electron in a nanowire with N_d_ dots in the effective mass approximation. The confining potential consists of N_d_ finite cylindrical potential wells of depth *V*, height *h*, and radius *a* (dots), separated by distance *D*, embedded in an infinite cylindrical well of length *L* and radius *r* (the wire) as shown in Fig. 1a,b. The potential well depth *V* is determined by the conduction band offset between strained InAs and InP, of the order of 100 meV^24^. The tunnelling barrier between two dots is determined by the potential depth V and barrier thickness controlled by the separation between InAs dots, of the order of nanometers. We expand the one-electron wavefunction ${\varphi }_{i}(\mathop{r}\limits^{\longrightarrow})$, where $\mathop{r}\limits^{\longrightarrow}$ is the position of electron in a nanowire, in terms of the eigenstates of the nanowire without quantum dots, products of Bessel functions in radial direction and trigonometric functions in the nanowire growth direction. We construct a one-electron Hamiltonian matrix in this basis and diagonalize it to obtain the one-electron spectrum *ε*(*iσ*) and eigenstates ${\varphi }_{i}(\vec{r})$. With ${c}_{i\sigma }^{+}$ (*c*_*iσ*_) the electron creation (annihilation) operators on the orbital *i* and spin *σ* the Hamiltonian of N_e_ electrons in a quantum dot array may be written as:

1 $$H=\sum _{i,\sigma }\varepsilon (i,\sigma ){c}_{i,\sigma }^{+}{c}_{i,\sigma }+\frac{1}{2}\sum _{ijkl,\sigma ,\sigma \text{'}}\langle i,j|{V}_{c}|k,l\rangle {c}_{i,\sigma }^{+}{c}_{j,\sigma \text{'}}^{+}{c}_{k,\sigma \text{'}}{c}_{l,\sigma }.$$

The first term is the energy of noninteracting electrons which captures the shell structure of individual dots and interdot tunneling. The second term, 〈*i*, *j*|*V*_*C*_|*k*, *l*〉, measured in effective Rydbergs, describes the Coulomb scattering of pairs of electrons from states *k,l* to states *i*, *j*, $\langle i,j|{V}_{c}|k,l\rangle =\iint d\mathop{{r}_{1}}\limits^{\longrightarrow}d\mathop{{r}_{2}}\limits^{\longrightarrow}{\varphi }_{i}^{\ast }(\mathop{{r}_{1}}\limits^{\longrightarrow}){\varphi }_{j}^{\ast }(\mathop{{r}_{2}}\limits^{\longrightarrow})\frac{2}{|{\overrightarrow{r}}_{1}-{\overrightarrow{r}}_{2}|}{\varphi }_{k}(\mathop{{r}_{2}}\limits^{\longrightarrow}){\varphi }_{l}(\mathop{{r}_{1}}\limits^{\longrightarrow})$. We compute Coulomb matrix elements using vectorized real-space discrete integration over orbitals and Coulomb interaction, with length and energy measured in effective Bohr radius ${{a}^{\ast }}_{B}=(\varepsilon /{m}^{\ast }){a}_{B}$ and effective Rydberg, Ry* = (m*)(1/*ε*)^2^ *Ry*. Here m* is the electron effective mass, *ε* is the dielectric constant, and *a*_*B*_ and *Ry* are the Bohr radius and Rydberg. In what follows we use *ε* = 12.4 and m* = 0.054 in the units of vacuum permittivity and free electron mass, respectively.

## Numerical results for one and two quantum dots

We illustrate our theory with numerical results for typical quantum dots in a nanowire, with *a* = r = 18 nm, *h* = 4 nm, *D* = 11 nm and *V* = 100 meV. For a single quantum dot confined in a nanowire we find characteristic s, p and d electronic shells. Populating this quantum dot with N_e_ = 4 electrons and diagonalizing the many-electron Hamiltonian, Eq. 1., in the space of configurations on the s, p and d-shells confirms the triplet ground state with total spin one obtained previously for quantum dots with parabolic lateral confinement^28–33^.

**Figure 2:**
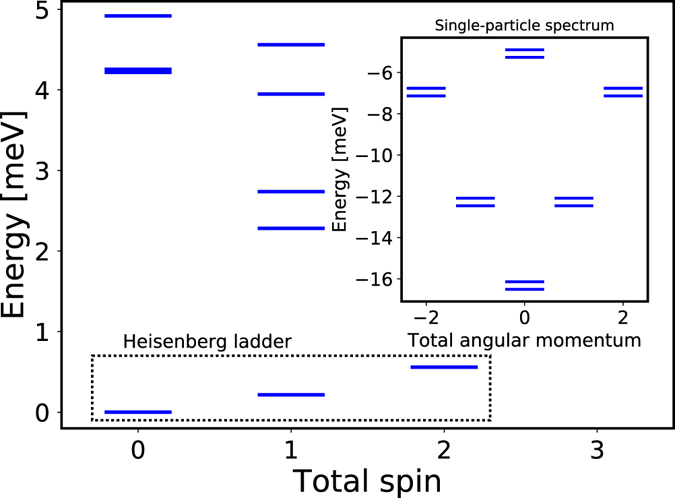
Low energy spectrum as a function of total spin S of N_e_ = 8 electrons on two quantum dots. The lowest energy levels in total S = 0,1,2 subspace are very well reproduced by the energy spectrum of the Heisenberg Hamiltonian of two spin ones coupled anti-ferromagnetically. The low energy states are isolated by an energy gap from higher energy excitations. Inset shows the single-particle energy spectrum showing hybridization of s,p, and d orbitals of each dot. Numerical results describe two quantum dots with height h = 4 nm, diameter a = r = 18 nm and separated by D = 11 nm.

We next determine the interaction of electrons in two quantum dots in a nanowire. With a basis of s-, p- and d-shells in each dot we expect orbitals with the same angular momentum to hybridize^34, 35^. Such a hybridized, numerically calculated single-particle spectrum is shown in the inset of Fig. 2. Indeed, for a double quantum dot we see a characteristic quasi-2D cylindrical shell structure with a splitting of each pair of angular momentum levels. The splitting is the difference in energy between the symmetric and anti-symmetric combination of angular momentum states on each dot^35^. The magnitude of the splitting, 2t, depends on the separation of dots *D* and barrier height *V*. We now populate the single particle spectrum of a double dot shown in Fig. 2 with N_e_ = 8 electrons, i.e., N_e_ = 4 electrons in each dot. Figure 2 shows the low-energy spectrum of N_e_ = 8 electrons in a double dot for different total spin S obtained using exact diagonalization techniques^1, 2, 28, 29, 33^. We find that the ground state has total spin S = 0, and the three lowest energy S = 0,1,2 states are very well separated from the higher energy states. To show that the singlet ground state of a double dot can be interpreted as antiferromagnetically coupled spin one states of each dot, let us compare the numerically computed electronic spectrum with a spectrum of a Heisenberg Hamiltonian for two spins one, $H=J\,{\mathop{S}\limits^{\longrightarrow}}_{1}\cdot {\mathop{S}\limits^{\longrightarrow}}_{2}$, and show that J > 0, i.e., the exchange coupling is antiferromagnetic. The spectrum of two spins one consists of an S = 0 singlet with energy E = −2J, S = 1 triplet at E = −J, and an S = 2 quintuplet at E =  + J. We compare these levels with the lowest three levels (not counting degenerate states) of the numerically obtained N_e_ = 8 electron spectrum by fitting J. These lowest levels are marked with the dotted box “Heisenberg ladder” in Fig. 2. They are indistinguishable from the spectrum of two interacting Heisenberg spins one on this energy scale. The agreement depends on the distance of the two dots, for large distance the tunneling induced splitting of the p-shell is smaller than the s-p-d shell separation. For short distances the concept of two weakly coupled p-shells is no longer valid and the N_e_ = 8 electrons populate a more complex shell structure of a double dot molecule. We can estimate the exchange interaction between electrons on the two dots as *J* = 2*t* ^2^/*U*. Taking the shell energy spacing as *ω*_0_, shell splitting by tunneling 2*t* = *ω*_0_, Coulomb repulsion on p-orbital as $U=0.6875\sqrt{\pi }\sqrt{{\omega }_{0}}$ ^28, 29^ we obtain $J\approx 0.41\,{\omega }_{0}^{3/2}\,Ry$. For a typical shell energy spacing of *ω*_0_ = 4 Ry we find an upper limit on the exchange coupling J~4 Ry~20 meV, comparable to room temperature.

## The spin one chain model

With Heisenberg model for two quantum dots established, we proceed to approximate the Hamiltonian for an array of N_d_ quantum dots with N_e_ = 4 electrons per dot, Eq. 1, by a Heisenberg Hamiltonian of a spin-one chain:

2 $$H=J\sum _{i=1}^{N-1}{\mathop{S}\limits^{\longrightarrow}}_{i}\,{\mathop{S}\limits^{\longrightarrow}}_{i+1}+g\mu {B}_{bg}{\mathop{S}\limits^{\longrightarrow}}_{tot}^{z}+g\mu {B}_{{1}}{\mathop{S}\limits^{\longrightarrow}}_{1}^{z}.$$

The first term describes antiferromagnetic interaction of nearest neighbour spins with strength J. The second term describes the effect of a homogeneous background magnetic field B_bg_ on a total spin ${\mathop{S}\limits^{\longrightarrow}}_{tot}^{z}=\sum _{i=1}^{N}{\mathop{S}\limits^{\longrightarrow}}^{{z}_{i}}$ from, e.g., a ferromagnetic substrate. The last term describes the local Zeeman coupling of a micromagnet with electron spins on a first site. Here *g* is the Lande factor and *μ* is the Bohr magneton.

**Figure 3:**
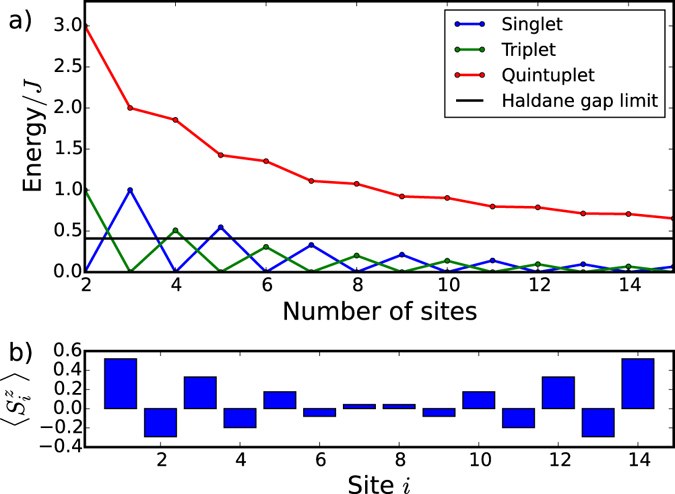
(**a**) Low energy spectrum of finite spin-one Heisenberg chains of N_d_ sites. Singlet, triplet and quintet states are shown, with singlet and triplet separated by the Haldane gap from the higher energy states. The Haldane gap limit of ~0.41 J^15^ is shown. (**b**) The expectation value of S_z_ in the triplet |*T*_+_〉 ground state at each quantum dot for 14-site chain. The edge states with S_z_ = 1/2 appear.

We first discuss the spectrum of a spin chain in the absence of external magnetic fields following previous work^11–17^. The ground state of an infinite chain is 4-fold degenerate, consisting of a degenerate total spin S = 0 singlet and total spin S = 1 triplet states, 4 states total, separated from excitations by an energy gap of *ΔE* = 0.41*J* ^10–16^. This is indeed shown in Fig. 3, which shows the energy spectrum of a finite spin-one chain, Eq. 2, obtained using the Lanczos method. While the spectrum for a spin-one chain of length N contains 3^N^ levels, only the low-energy spectrum is shown. The total spin of each energy level is determined from its degeneracy. We show the singlet S = 0 levels in blue, S = 1 triplet levels in green and S = 2 quintet levels in red colours. We see that the singlet and triplet energy levels are separated by an energy gap from quintet and higher energy states. The energy gap approaches 0.41 J with increasing chain length. The singlet and triplet alternate as ground states, and their energy splitting decreases to zero with increasing chain length. The singlet-triplet energy structure of the ground state can be understood with effective spin-1/2 quasiparticles localized at the end of the chain and interacting over the macroscopic distance. Figure 3b shows the calculated expectation value of the spin on the i-th site along the chain, $\langle GS|{\mathop{S}\limits^{\longrightarrow}}^{{z}_{i}}|GS\rangle$, for the triplet ground state |*T*_+_〉. Indeed, despite the fact that each non-interacting spin can have only $\langle {\mathop{S}\limits^{\longrightarrow}}^{{z}_{i}}\rangle =0,\pm 1$, the expectation value of the spin in the ground state |*GS*〉 of the interacting chain corresponds to two spin-1/2 quasiparticles localized at the end of the chain. These quasiparticles are coupled with each other over macroscopic wire length, with the coupling decreasing with length. Note that since the Haldane gap is of the order of the exchange constant, the upper limit on the Haldane gap is also comparable to the room temperature.

## Haldane spin-1/2 quasiparticles and a spin singlet-triplet qubit

**Figure 4:**
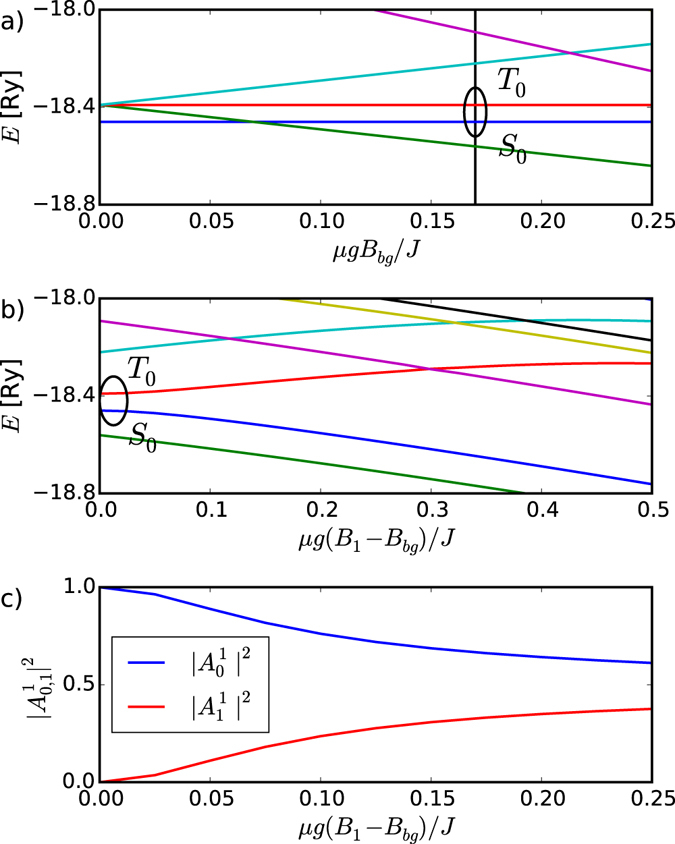
Isolating and operating a singlet-triplet qubit in a spin one chain. (**a**) isolating two singlet-triplet qubit levels |*S*_0_〉 and |*T*_0_〉 of a 14-site spin one chain with increasing uniform background magnetic field B_bg_ (**b**) rotating the singlet-triplet qubit by magnetic field B_1_ applied to first quantum dot at a fixed B_bg_, (**c**) evolution of probability density of the qubit states in the rotated qubit levels shown in (**b**). In all the subfigures, blue and red lines denote |*S*_0_〉 and |*T*_0_〉 states, respectively.

We now turn to define a singlet-triplet qubit^1–4, 32^ using the spin-1/2 quasiparticles of the spin-one chain. In a chain of even number N_d_ of quantum dots, the singlet ground state |S_0_〉 has total spin S = 0, and there are 3 excited states |*T*_0_〉, |*T*_+_〉|*T*_−_〉 of the threefold degenerate spin-one triplet manifold, with total S_z_ = 0, + 1, −1. To isolate two qubit states we apply the finite magnetic field B, measured in units of the ratio of the Zeeman to exchange energy *gμB*/*J*. In Fig. 4a, we plot the evolution of the numerically obtained low energy spectrum of N_d_ = 14 spins one, Eq. 2. There are ~5 million states in total; we only show the evolution of the lowest few energy levels. We see that the |*T*_+_〉 and |*T*_−_〉 energy levels split from the two, |*T*_0_〉 and |*S*_0_〉 levels selected as the two qubit levels. These two levels are split by exchange energy, J_2_, giving effective magnetic field along z-axis. We want this splitting to be finite, but much lower than separation of these states from the rest. Therefore we expect that the chain length of N_d_ = 14 is close to optimal for the qubit performance. We also see that at high applied magnetic fields the energy of the quintet |*S* = 0, *S*_*z*_ = −2〉 level starts approaching the two S_z_ = 0 qubit levels and we must optimize the qubit-quintet energy gap. To implement the rotation of a qubit state, we create a linear superposition $|{a}_{i}\rangle ={A}_{0}^{i}|{S}_{0}\rangle +{A}_{1}^{i}|{T}_{0}\rangle$ of the |T_0_ 〉 and |S_0_ 〉 qubit levels by applying a local magnetic field B_1_ to the first dot. Because the singlet and triplet are entangled states of two spin-1/2 quasiparticles, acting on only one of them rotates the whole state. The evolution of the low-lying levels for homogeneous external field *gμB*/*J* = 0.17, for which the qubit levels are well separated from the quintet levels, as a function of the local magnetic field B_1_ is shown in Fig. 4b. We see that increasing the local field B_1_ results in the evolution of the two qubit levels |*a*_0_〉 and |*a*_1_〉. To verify that we indeed rotate the qubit we calculate the projections $|\langle {S}_{0}|{a}_{1}\rangle {|}^{2}=|{A}_{0}^{1}{|}^{2}$ and $|\langle {T}_{0}|{a}_{1}\rangle {|}^{2}=|{A}_{1}^{1}{|}^{2}$ of the exact numerically calculated qubit eigenstates |*a*_*i*_〉. The result, confirming rotation of the qubit, is shown in Fig. 4c. We note that the isolation of qubit states from higher excitations due to the Haldane gap makes the admixture of higher energy excitations into the qubit levels very small, at most 1.2% (see Fig. 4).

## Discussion

The macroscopic singlet-triplet qubit proposed here has several advantages compared to other, spin, charge and superconducting qubits. Most of all it is macroscopic and yet semiconductor based. It hence combines advantages of macroscopic superconducting and microscopic semiconductor qubits^1, 2^. Being macroscopic, large, and semiconductor makes it easier to fabricate, control and integrate with existing semiconductor based microelectronics. With electrons confined in self-assembled quantum dots with band offsets of the order of 100 meV it is stable at much higher temperature than electron spin based qubits in lateral gated quantum dots, refs 1, 2 where confining potential is of the order of 1 meV. The qubit is also robust against fluctuations in exchange coupling due to composition and interdot distance variations as shown in ref. 36. The states of a qubit can be probed optically as both photo-excited electrons and holes are located in the InAs quantum dots containing electrons building effective spin-1/2 quasiparticles^24, 25^. If nanowires are fabricated using InAs in InP, the qubit states can be converted to photons in the telecom range. If decoherence due to nuclear spins in the InAs/InP system turns out to be a problem, the qubit can be fabricated in Si/SiGe system^37, 38^. Such robust qubits, once demonstrated, can in principle be assembled into a quantum circuit as the interaction of capacitively coupled singlet-triplet qubits in lateral gated quantum dots has been already demonstrated^39^.

## Conclusions

In summary, we proposed here a synthetic spin-one chain in a semiconductor nanowire realizing macroscopic quantum states in a semiconductor device. The synthetic spin chain is created by quantum dots in a nanowire with four electrons each. Using exact diagonalization techniques we show that the ground state of each dot corresponds to spin one and the coupling between spins on each dot is anti-ferromagnetic. We show that the low energy states of the macroscopic wire correspond to two quasiparticles with spin ½ localised at the ends of the nanowire. Using a homogeneous magnetic field and a micromagnet at one end we show how to define and operate a robust, macroscopic singlet-triplet qubit, protected from decoherence by a Haldane gap. The gap, determined by anti-ferromagnetic coupling J, has the potential to reach room temperature. Future work will focus on theory of interaction of synthetic spin-one chain with light, hyperfine coupling with nuclear spins, microscopic nature of capacitive coupling of two qubits, effects of unintentional variation in quantum dot size and separation as well as its realisation in Si/SiGe material system.

### Data availability statement

The datasets generated during and/or analysed during the current study are available from the corresponding author on reasonable request.
